# Correlation between endometriosis and chronic endometritis: A retrospective cohort study

**DOI:** 10.1097/MD.0000000000051450

**Published:** 2026-10-30

**Authors:** HuiLiu Fan, Bei Liu, XiaoXia Wu, HongLan Wei

**Affiliations:** a Department of Gynaecology, Guangzhou Women and Children’s Medical Center Liuzhou Hospital, Liuzhou, Guangxi, China; b Guangxi Clinical Research Center for Obstetrics and Gynecology, Liuzhou, Guangxi, China.

**Keywords:** chronic endometritis, endometriosis, hysteroscopy, infertility, rASRM, recurrent miscarriage

## Abstract

This single-center retrospective cohort study included infertile patients treated between October 2019 and December 2024, comparing an endometriosis (EMS) cohort (n = 250, laparoscopically and histopathologically confirmed) with a control cohort (n = 750, tubal-factor infertility only, EMS excluded by laparoscopy). All patients underwent laparoscopy, hysteroscopy, and endometrial biopsy; chronic endometritis (CE) was diagnosed by cluster of differentiation 38/cluster of differentiation 138 immunohistochemistry, and EMS was staged by revised American Society for Reproductive Medicine. CE detection rate was significantly higher in the EMS cohort than in controls (*P* < .001). Within the EMS cohort, CE prevalence was 45.1% in moderate-to-severe (stage III–IV) disease versus 24.0% in mild (stage I–II) disease (*P* = .001). Factors independently associated with concurrent CE were moderate-to-severe EMS, repeated intrauterine procedures, infertility duration ≥ 3 years, chronic pelvic pain, and higher gravidity (all *P* < .05). At 1 year, the EMS cohort had a lower clinical pregnancy rate (70.0% vs 80.0%), higher persistent infertility rate (30.0% vs 20.0%), and higher early pregnancy loss rate (16.0% vs 9.2%) than controls (all *P* < .05). Within the EMS cohort, concurrent CE was associated with a lower clinical pregnancy rate (56.0% vs 76.0%), higher persistent infertility rate (44.0% vs 24.0%), and higher early pregnancy loss rate (31.0% vs 11.3%) than EMS alone (all *P* = .002). These findings demonstrate a significant cross-sectional association between EMS and CE, with higher CE detection in advanced-stage EMS; however, causality and temporal sequence cannot be determined, reproductive outcomes were exploratory and unadjusted, and this single-center retrospective dataset is insufficient to support universal diagnostic or therapeutic guidelines.

## 1. Introduction

Endometriosis (EMS) and chronic endometritis (CE) are prevalent chronic gynecological conditions affecting women of reproductive age. Both are major contributors to chronic pelvic pain, infertility, and early pregnancy loss, imposing a substantial burden on reproductive health and quality of life.^[1]^

EMS is a chronic inflammatory disorder characterized by the presence of ectopic endometrial glands and stroma outside the uterine cavity. Although estrogen promotes the proliferation of ectopic lesions and exacerbates pelvic pain, EMS is not strictly estrogen-dependent. Clinical evidence shows that gonadotropin-releasing hormone agonists effectively alleviate EMS-related pelvic pain but rarely eradicate established ectopic lesions. Moreover, ectopic endometrial lesions have been reported in prepubertal individuals and male patients-populations with extremely low circulating estrogen levels-confirming that estrogen alone cannot fully account for disease pathogenesis.^[2,3]^ EMS disrupts pelvic immune homeostasis and intra-abdominal endocrine signaling, generating a sustained pro-inflammatory pelvic microenvironment.^[3]^

CE is defined as persistent low-grade endometrial inflammation, pathologically characterized by cluster of differentiation (CD)38 + and CD138 + plasma cell infiltration, accompanied by intrauterine immune dysregulation and impaired mucosal barrier function.^[4,5]^ Despite their distinct primary lesion sites, EMS and CE share overlapping inflammatory pathways and aberrant sex hormone signaling, suggesting potential biological interplay between the 2 conditions.^[6]^

Previous observational studies have reported markedly elevated CE detection rates in EMS patients, with meta-analyses yielding pooled prevalence estimates up to 53%.^[6]^ Approximately 56.8% of infertile women are affected by CE, and abundant clinical data have confirmed strong associations between CE and adverse reproductive outcomes, including recurrent miscarriage and repeated implantation failure.^[7]^ Consequently, the coexistence of EMS and CE and its potential impact on fertility has attracted growing clinical interest.

Conventional gynecological practice typically prioritizes the diagnosis and management of a single pelvic disorder, lacking standardized simultaneous evaluation for both EMS and CE. CE presents with mild, nonspecific symptoms that are easily masked by classic EMS features such as progressive dysmenorrhea and pelvic discomfort, leading to underdiagnosis of concurrent pathology. This oversight may result in missed dual diagnoses, incomplete therapeutic intervention, elevated recurrence risk, and suboptimal reproductive outcomes.

This retrospective cohort study quantified the observational association between EMS and CE, identified clinical variables correlated with their coexistence, and explored their combined impact on reproductive outcomes. The findings provide preliminary data to optimize clinical assessment strategies for infertile patients with EMS and offer directions for future therapeutic approaches.

## 2. Materials and methods

### 2.1. Study design and participants

This single-center retrospective observational cohort study was conducted using electronic medical records from our institution, covering data from October 2019 to December 2024. All clinical data were retrospectively extracted from institutional medical archives and fully de-identified prior to analysis to protect patient confidentiality. No prospective participant recruitment was performed. This study was approved by the Institutional Ethics Committee (Approval No. 2026-162). This work was supported by the Guangxi Science and Technology Plan Project (Guangxi Clinical Research Center for Obstetrics and Gynecology, Grant No. GuiKe AD22035223).

The EMS cohort comprised 250 infertile women aged 20 to 45 years with laparoscopically visualized ectopic lesions and histopathologically confirmed EMS. The control cohort included 750 age-matched infertile women aged 20 to 45 years whose infertility was attributed exclusively to tubal pathology. All control patients underwent diagnostic laparoscopy as part of their infertility workup, which excluded laparoscopic or histologic evidence of EMS or other organic pelvic lesions; subtle or microscopic EMS, however, could not be entirely excluded (see Limitations). All patients in both cohorts underwent standardized hysteroscopy and endometrial biopsy during the study period.

### 2.2. Diagnostic criteria

EMS diagnosis required both characteristic laparoscopic macroscopic lesions and histopathological confirmation of ectopic endometrial tissue. Intraoperative staging was performed using the 1997 revised American Society for Reproductive Medicine (rASRM) scoring system: Stage I (minimal, 1–5 points); Stage II (mild, 6–15 points); Stage III (moderate, 16–40 points); Stage IV (severe, > 40 points).

CE diagnosis was based on immunohistochemical testing of endometrial biopsy specimens, which served as the reference standard, combined with hysteroscopic morphological assessment. The operational diagnostic algorithm was as follows: all patients underwent hysteroscopy, and morphological findings suggestive of CE (e.g., micropolyps, diffuse hyperemia, stromal edema) were recorded; endometrial biopsy specimens were immunohistochemically stained for CD38 and CD138; and CE was diagnosed when ≥ 5 CD38+/CD138 + plasma cells were identified in 30 randomly selected high-power fields (×400), consistent with published diagnostic criteria,^[8]^ irrespective of the hysteroscopic appearance. Thus, immunohistochemistry alone determined the final CE status. High-power fields were selected in a randomized manner across representative areas of each specimen, avoiding necrotic or hemorrhagic regions. All pathology slides were reviewed independently by 2 experienced gynecological pathologists who were blinded to the patients’ EMS status, and discrepant interpretations were resolved by consensus.

### 2.3. Inclusion criteria

All patients met the following criteria:

Aged 20 to 45 years;

Primary diagnosis of infertility with complete fertility workup;

Diagnostic laparoscopy for both cohorts (to confirm and stage EMS in the EMS cohort, and to exclude EMS as part of the tubal-factor infertility workup in the control cohort); all patients underwent hysteroscopy and endometrial biopsy;

Complete electronic medical records with baseline data, medical history, intraoperative findings, pathology results, postoperative treatment, and 1-year reproductive outcome data.

### 2.4. Exclusion criteria

Patients were excluded for:

1. Acute reproductive tract infections or other organic uterine/ovarian lesions (e.g., leiomyomas, adenomyosis, polyps, adhesions, hyperplasia, malignancies);
2. Use of systemic antibiotics, sex hormones, or immunosuppressants within 3 months before endoscopy; prior hysterectomy or confirmed endometrial tuberculosis;
3. Autoimmune diseases, severe hepatic/renal dysfunction, or hematologic malignancies; incomplete medical records; failure to complete hysteroscopy and biopsy;
4. Severe diminished ovarian reserve or male-factor infertility identified in pretreatment fertility screening, to minimize confounding of baseline fertility potential.

### 2.5. Data extraction and postoperative management

Trained researchers extracted standardized data from electronic medical records, including age, body mass index (BMI), gravidity, parity, history of intrauterine procedures, infertility duration, chronic pelvic pain, rASRM stage, hysteroscopic findings, CE diagnosis, postoperative treatment, and 1-year reproductive outcomes. No prospective data collection or patient contact was performed.

Per institutional protocols, patients with EMS received 3 months of gonadotropin-releasing hormone agonists or oral dydrogesterone after laparoscopy.^[1]^ Patients with confirmed CE received a 14-day course of oral antibiotics based on expert consensus.^[9]^ The 1-year reproductive follow-up started on the date of the index laparoscopic procedure for all patients. Because the duration of postoperative medical therapy differed between conditions (3 months of hormonal therapy for EMS vs 14 days of antibiotics for CE), the effective time at risk for conception may have differed slightly between groups; this is acknowledged in the Limitations. Early pregnancy loss rate was calculated as: (number of early miscarriages during follow-up) ÷ (total clinical pregnancies) × 100%. Pregnancies were not stratified by conception mode (spontaneous vs assisted reproductive technology), which is acknowledged as a limitation.

### 2.6. Statistical analysis

Statistical Package for the Social Sciences 22.0 (IBM Corp.) was used for all analyses. Continuous variables (age, BMI, gravidity, parity) were expressed as mean ± standard deviation and compared using independent-samples t-tests. Categorical variables (CE status, reproductive outcomes) were presented as counts (percentages) and compared using Pearson chi-squared tests. The multivariable binary logistic regression was restricted to the 250 women with EMS, with CE status as the dependent variable (CE = 1, no CE = 0). Candidate predictors included age, BMI, gravidity, parity, rASRM stage (III–IV vs I–II), history of repeated intrauterine procedures (≥ 2 vs < 2), infertility duration (≥ 3 vs < 3 years), and chronic pelvic pain (present vs absent). Age, BMI, gravidity, and parity were entered as continuous variables, whereas rASRM stage, repeated intrauterine procedures, infertility duration, and chronic pelvic pain were entered as binary variables. The cut-points for infertility duration (≥ 3 years) and repeated intrauterine procedures (≥ 2) were selected on the basis of clinical convention and the observed distribution of the study data. Because the choice of these thresholds was informed by the study data, they were not prespecified and are best regarded as data-derived. All candidate predictors were entered simultaneously into the model (enter method). The complete model, including predictors that were not statistically significant, is reported, together with β coefficients and odds ratios (OR) with 95% confidence intervals (CI). Multicollinearity among predictors was assessed using variance inflation factors, and model calibration was evaluated with the Hosmer–Lemeshow goodness-of-fit test; the corresponding results are reported in Section 3.4. Because complete baseline and outcome data were required by the inclusion criteria, no imputation of missing data was necessary. All tests were 2-tailed, with *P* < .05 considered statistically significant.

## 3. Results

### 3.1. Baseline characteristics

A total of 1245 infertile women aged 20 to 45 years were screened during the study period, of whom 245 were excluded for the following reasons: reproductive tract infection, uterine or ovarian organic lesions, recent antibiotic/hormone/immunosuppressant use, comorbid internal medical diseases, severe hepatic/renal dysfunction, severe diminished ovarian reserve, or male-factor infertility (n = 183); and incomplete medical records or incomplete 1-year follow-up (n = 62). After applying the inclusion and exclusion criteria, 250 EMS patients were assigned to the observation cohort and 750 tubal infertility patients to the control cohort; complete baseline and outcome data were available for all included patients. No statistically significant differences were observed between cohorts in age, BMI, gravidity, or parity (all *P* > .05; Table 1), and the absolute differences were small (e.g., 0.36 years in mean age and 0.16 kg/m^2^ in mean BMI) and were judged unlikely to be clinically meaningful. Nevertheless, because of the observational design, statistically nonsignificant tests cannot establish baseline equivalence or exclude residual confounding (Fig. 1)

**Table 1:** Comparison of general data between the 2 groups ($\bar{x}\pm \;s$).

Group	n	Age (yrs)	BMI (kg/m^2^)	Gravidity	Parity
Observation	250	31.25 ± 4.16	22.86 ± 2.31	1.52 ± 0.68	0.41 ± 0.35
Control	750	30.89 ± 4.32	23.02 ± 2.45	1.48 ± 0.72	0.38 ± 0.32
*t*	-	1.217	0.866	0.764	1.202
*P*	-	.224	.386	.445	.230

BMI = body mass index, n = number of participants.

**Figure 1.**
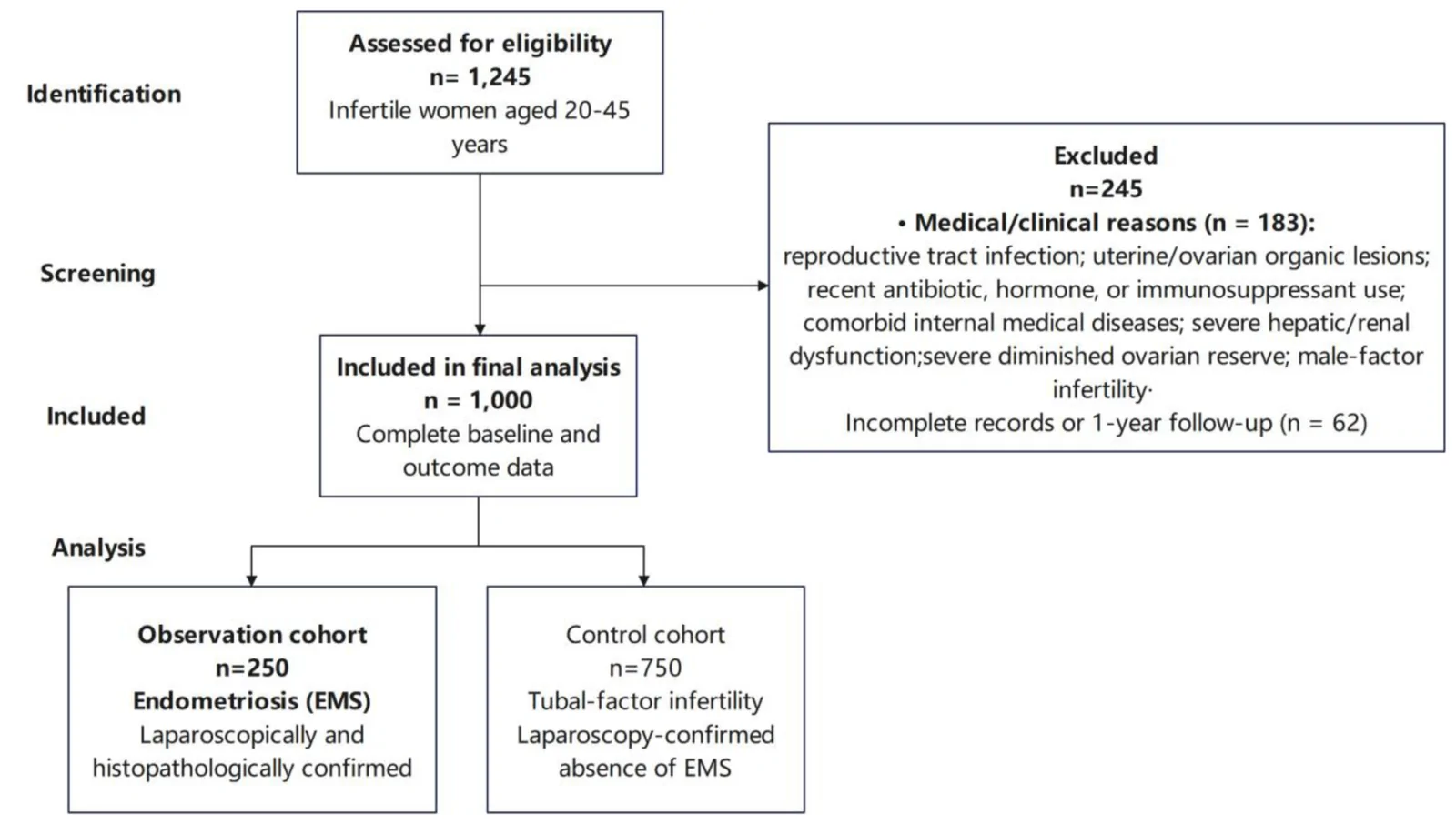
Flow diagram of participant inclusion and exclusion. Of 1245 screened women, 245 were excluded (183 for medical/clinical reasons; 62 for incomplete records or 1-year follow-up), leaving 1000 for analysis (250 EMS observation cohort vs 750 tubal-factor infertility control cohort). EMS = endometriosis, n = number of participants.

### 3.2. CE detection rates

CE was confirmed in 75 of 250 EMS patients (30.0%), compared with 120 of 750 control patients (16.0%), a statistically significant difference (*χ*^2^ = 23.411, *P* < .001). Detailed data are presented in Table 2.

**Table 2:** Comparison of the prevalence of CE between the 2 groups.

Group	n	CE cases	CE prevalence, n (%)	*χ* ^2^	*P*
Observation (EMS)	250	75	30.0	23.411	< .001
Control	750	120	16.0	-	-

CE = chronic endometritis, EMS = endometriosis, n = number of participants.

### 3.3. CE prevalence by rASRM stage

Among 179 patients with mild (stage I–II) EMS, 43 (24.0%) had CE. Among 71 patients with moderate-to-severe (stage III–IV) EMS, 32 (45.1%) had CE. CE prevalence was significantly higher in the moderate-to-severe group (*χ*^2^ = 10.724, *P* = .001). Stage-specific data are shown in Table 3.

**Table 3:** Comparison of CE prevalence in patients with different stages of EMS.

Group	n	CE cases	CE prevalence, n (%)	*χ* ^2^	*P*
Mild EMS (stage I–II)	179	43	24.0	10.724	.001
Moderate-to-severe EMS (stage III–IV)	71	32	45.1	-	-

CE = chronic endometritis, EMS = endometriosis, n = number of participants.

### 3.4. Factors associated with concurrent EMS and CE

Multivariable logistic regression restricted to the 250 patients with EMS, with CE status as the outcome, included all 8 candidate predictors (age, BMI, gravidity, parity, rASRM stage, history of repeated intrauterine procedures, infertility duration, and chronic pelvic pain); the complete model, including predictors that were not statistically significant, is presented in Table 4. Five factors were independently associated with concurrent EMS and CE (*P* ≤ .001 for all 5): moderate-to-severe (rASRM stage III–IV) EMS, history of repeated intrauterine procedures, infertility duration ≥ 3 years, chronic pelvic pain, and higher gravidity; the remaining 3 predictors (age, BMI, and parity) were not statistically significant. Multicollinearity diagnostics showed a maximum variance inflation factor of 1.04, indicating no evidence of multicollinearity. Model calibration was assessed with the Hosmer–Lemeshow goodness-of-fit test (*χ*^2^ = 3.540, df = 8, *P* = .896), indicating good model fit. β coefficients, ORs, 95% CIs, and *P* values for all 8 predictors are presented in Table 4.

**Table 4:** Multivariate logistic regression analysis of risk factors for EMS complicated with CE (analytical population: the 250 patients with EMS; dependent variable: CE status).

Risk factor	*β*	SE	OR	95% CI	*P*
Moderate-to-severe EMS (stage III–IV vs I–II)	1.263	0.214	3.53	2.32–5.37	< .001
Repeated intrauterine procedures (≥ 2 vs < 2)	0.987	0.196	2.68	1.83–3.92	< .001
Infertility duration (≥ 3 yrs vs < 3 yrs)	0.852	0.182	2.34	1.64–3.34	< .001
Chronic pelvic pain (present vs absent)	0.794	0.175	2.21	1.57–3.11	< .001
Age (yrs)	0.007	0.034	1.01	0.94–1.08	.848
BMI (kg/m^2^)	0.033	0.061	1.03	0.92–1.17	.587
Gravidity	0.778	0.243	2.18	1.35–3.51	.001
Parity	0.305	0.270	1.36	0.80–2.30	.260

The dependent variable was concurrent CE in the 250 patients with EMS; all 8 candidate predictors were entered simultaneously into the model, and predictors that were not statistically significant are reported. Multicollinearity was assessed using variance inflation factors and model calibration with the Hosmer–Lemeshow goodness-of-fit test (Section 3.4). Five of the 8 predictors were statistically significant (*P* ≤ .001 for all 5). For the continuous predictors (age, BMI, gravidity, and parity), ORs correspond to a 1-unit increase; for the binary predictors, ORs correspond to the contrast given in parentheses.

β: regression coefficient.

CE = chronic endometritis, CI = confidence interval, EMS = endometriosis, OR = odds ratio, SE = standard error, VIF = variance inflation factor.

### 3.5. Reproductive outcomes at 1 year

After standardized treatment, the EMS cohort had a lower clinical pregnancy rate (175/250, 70.0% vs 600/750, 80.0%; *χ*^2^ = 10.753, *P* = .001), a higher rate of persistent infertility (75/250, 30.0% vs 150/750, 20.0%; *P* = .001), and a higher early pregnancy loss rate among clinical pregnancies (28/175, 16.0% vs 55/600, 9.2%; *χ*^2^ = 6.616, *P* = .010) than the control cohort. In the subgroup analysis within the EMS cohort (Table 5), patients with concurrent CE had a significantly lower clinical pregnancy rate (56.0%, 42/75) than those without CE (76.0%, 133/175; *χ*^2^ = 10.000, *P* = .002), a higher rate of persistent infertility (44.0%, 33/75 vs 24.0%, 42/175; *χ*^2^ = 10.000, *P* = .002), and a higher early pregnancy loss rate among clinical pregnancies (31.0%, 13/42 vs 11.3%, 15/133; *χ*^2^ = 9.193, *P* = .002).

**Table 5:** Comparison of adverse pregnancy outcomes between the study and control groups, with subgroup analysis by concurrent CE status within the EMS cohort.

Group	n	Pregnancy rate, n (%)	Infertility rate, n (%)	Early pregnancy loss rate, n (%)
Observation (EMS), overall	250	175 (70.0)	75 (30.0)	28 (16.0)
EMS without CE	175	133 (76.0)	42 (24.0)	15 (11.3)
EMS with CE	75	42 (56.0)	33 (44.0)	13 (31.0)
Control	750	600 (80.0)	150 (20.0)	55 (9.2)
*χ*^2^ (EMS overall vs control)	-	10.753	10.753	6.616
*P* (EMS overall vs control)	-	.001	.001	.010
*χ*^2^ (EMS with CE vs without CE)	-	10.000	10.000	9.193
*P* (EMS with CE vs without CE)	-	.002	.002	.002

The pregnancy rate and the infertility rate were calculated using the total number of patients in each group as the denominator. The early pregnancy loss rate was calculated among clinical pregnancies, so its denominator (the number of clinical pregnancies in each group) differs from those of the other 2 outcomes: 28/175 (EMS overall), 15/133 (EMS without CE), 13/42 (EMS with CE), and 55/600 (control). Percentages are rounded to 1 decimal place.

CE = chronic endometritis, EMS = endometriosis, n = number of participants.

## 4. Discussion

### 4.1. Observational association between EMS and CE

This retrospective analysis found a 30.0% CE detection rate in EMS patients, significantly higher than the 16.0% rate in tubal infertility patients without EMS. These findings align with published meta-analyses and retrospective cohorts. Kalaitzopoulos et al^[10]^ reported a pooled CE prevalence of 28% in EMS patients, with rates of 43% in rASRM stage III–IV versus 25% in stage I–II disease (OR = 2.07, 95% CI = 1.11–3.84); Cicinelli et al^[11]^ observed CE in 38.5% of women with EMS versus 14.1% of controls on immunohistochemistry. In a meta-analysis of 4145 infertile women, Vitagliano et al^[12]^ likewise reported that CE was associated with lower ongoing-pregnancy/live-birth rates (OR = 1.97) and clinical pregnancy rates (OR = 2.28) after in vitro fertilization. Cumulative evidence supports a positive cross-sectional association between EMS and CE. However, the retrospective design precludes causal inference or determination of temporal sequence; co-occurrence may reflect bidirectional inflammatory crosstalk or unmeasured confounding.

Two potential bidirectional mechanisms have been proposed. First, chronic pelvic inflammation induced by EMS alters pelvic anatomy, causing tubal adhesions and hydrosalpinx. Reflux of inflammatory peritoneal fluid through the tubal ostia into the uterine cavity continuously stimulates the endometrium, promoting CE.^[10,13,14]^ At the molecular level, EMS sustains a pro-inflammatory pelvic microenvironment with elevated interleukin-6 and tumor necrosis factor-α. These cytokines diffuse into the uterine cavity, disrupt the endometrial mucosal immune barrier, and drive plasma cell infiltration characteristic of CE.^[10,15,16]^ Conversely, preexisting CE may aggravate retrograde menstruation and ectopic implantation, increasing EMS burden and forming a self-amplifying inflammatory feedback cycle.^[17]^ Additionally, shared confounding factors such as repeated intrauterine instrumentation, chronic pelvic inflammation, and varied gynecologic visit frequency may independently increase detection risks for both conditions.

Subgroup analysis revealed higher CE prevalence in moderate-to-severe EMS than in mild EMS, supporting the hypothesis that advanced EMS generates stronger systemic pelvic inflammation, leading to more pronounced disruption of intrauterine immune homeostasis and higher CE risk.^[18]^ These stratified findings underscore the inflammatory link between EMS and CE and identify high-risk subgroups for targeted clinical assessment.

### 4.2. Clinical correlates of concurrent EMS and CE

Multivariate regression identified 5 factors independently associated with concurrent EMS and CE: repeated intrauterine procedures, infertility duration ≥ 3 years, chronic pelvic pain, advanced EMS staging, and higher gravidity. Higher gravidity may partly reflect cumulative intrauterine exposure and repeated pregnancy-related instrumentation, and it remained associated with concurrent CE after adjustment for the other predictors. Repeated intrauterine interventions (e.g., induced abortion, diagnostic hysteroscopy, intrauterine device insertion/removal) directly disrupt endometrial integrity. Such procedures simultaneously increase retrograde menstruation risk, facilitating ectopic endometrial implantation and EMS progression,^[19,20]^ while damaging mucosal and microbial barriers, promoting ascending infection and subsequent CE.^[21,22]^

Prolonged infertility and chronic pelvic pain represent shared manifestations of superimposed inflammatory signaling from both EMS and CE.^[1]^ Sustained chronic inflammation progressively impairs pelvic organ function and endometrial receptivity. Ectopic EMS lesions continuously secrete pro-inflammatory cytokines (interleukin-6, tumor necrosis factor-α), triggering diffuse pelvic inflammation, tubal structural distortion, hydrosalpinx, ciliary injury, and impaired peristalsis, thereby disrupting normal tubo-ovarian function.^[6,23]^

Inflammatory exudate and tubal fluid reflux into the uterine cavity carry pathogenic bacteria and dysbiotic flora, 2 distinct but interrelated inflammatory triggers. Pathogenic bacteria represent discrete infectious species, while dysbiotic flora describes overall structural imbalance of the native endometrial microbiome. Both continuously stimulate endometrial tissue, disrupt local immune barrier function, induce CD138 + plasma cell infiltration, initiate and sustain chronic endometrial inflammation, and establish a self-perpetuating cycle of inflammatory injury and impaired fertility.^[10,24]^

Clinically, EMS typically presents with progressive dysmenorrhea and chronic pelvic pain, whereas CE often manifests with subtle nonspecific symptoms or remains asymptomatic. Prominent EMS symptoms mask mild CE manifestations, which is the primary reason for missed dual diagnoses. Clinicians should maintain high suspicion for coexisting CE in EMS patients presenting with the 5 identified risk factors to avoid incomplete therapeutic intervention.

### 4.3. Interpretation of reproductive outcomes

All patients received standardized postoperative treatment per institutional protocols. One-year follow-up data showed higher rates of persistent infertility and early pregnancy loss in the overall EMS cohort than in the control cohort, even after postoperative medical therapy. Plausible mechanisms linking EMS and concurrent CE to impaired reproduction include impaired endometrial receptivity, disrupted microbiome balance, pelvic anatomical distortion, and sustained inflammatory signaling, which may disturb ovulation, fertilization, implantation, and early embryonic development.^[25–28]^ In the subgroup analysis within the EMS cohort, women with concurrent CE showed a lower clinical pregnancy rate (56.0% vs 76.0%), a higher rate of persistent infertility (44.0% vs 24.0%), and a higher early pregnancy loss rate among clinical pregnancies (31.0% vs 11.3%) than those with EMS alone (all *P* = .002). However, because reproductive outcomes were not stratified by conception mode or EMS severity, and the subgroup comparison by concurrent CE status within the EMS cohort (Table 5) was unadjusted for potential confounders such as EMS stage, these findings should be interpreted as hypothesis-generating, and no causal inference regarding a combined adverse reproductive impact of concurrent EMS and CE can be drawn. Further prospective studies with prespecified, confounder-adjusted subgroup analyses are needed to optimize standardized medical treatment regimens for patients with concurrent EMS and CE.

From a clinical perspective, isolated surgical or hormonal intervention for EMS cannot resolve CE-mediated intrauterine inflammation, and CE-targeted antibiotic therapy cannot alleviate EMS-induced pelvic adhesions or extrauterine inflammatory signaling. These complementary therapeutic limitations support the clinical value of simultaneous evaluation for both disorders in high-risk EMS patients and the need for integrated therapeutic approaches addressing both conditions.

### 4.4. Study limitations

Several limitations should be acknowledged:

1. All subjects underwent surgical intervention, representing a symptomatic subgroup unresponsive to medical therapy; findings may not generalize to asymptomatic EMS patients managed conservatively.
2. The retrospective design precludes determination of temporal sequence or causal inference between EMS and CE. Unmeasured confounders (e.g., gynecologic surveillance frequency, prior pelvic infection, hormonal treatment exposure) may partially mediate the observed association.
3. This single-center dataset (250 EMS patients) lacks external validation and is insufficient to formulate broad screening recommendations. In addition, the multivariable model was fitted with 75 outcome events and 8 candidate predictors (approximately 9 events per variable), which is at the lower end of the conventionally recommended ratio; no variable selection was performed, and the estimates for predictors that were not statistically significant should therefore be regarded as exploratory and interpreted with caution.
4. Reproductive outcomes were not stratified by conception mode (spontaneous vs assisted reproductive technology). The subgroup comparison of reproductive outcomes by concurrent CE status within the EMS cohort (Table 5) was not adjusted for EMS stage or other potential confounders; although the observed between-subgroup differences were statistically significant, they should therefore be interpreted with caution. In addition, the differing duration of postoperative medical therapy (3 months of hormonal therapy for EMS vs 14 days of antibiotics for CE) may have resulted in unequal time at risk for conception between groups.
5. Although all control patients underwent diagnostic laparoscopy, subtle or microscopic EMS cannot be entirely excluded, and residual exposure misclassification cannot be ruled out.
6. Sixty-two screened women were excluded because of incomplete medical records or incomplete 1-year follow-up. Because the inclusion criteria required complete baseline and outcome data, the final analytic cohort was restricted to women with complete data, which may have introduced selection or attrition bias: women lost to follow-up may differ systematically from those retained with respect to disease severity, treatment adherence, or reproductive prognosis. Baseline data were not available for the excluded women, so a formal comparison of baseline characteristics between included and excluded participants could not be performed, and the direction and magnitude of any resulting bias cannot be quantified. These considerations should be taken into account when interpreting the findings.

## 5. Conclusion

This single-center retrospective analysis demonstrates a significant cross-sectional association between EMS and CE, with higher CE detection rates in moderate-to-severe EMS. Five factors (advanced EMS stage, repeated intrauterine procedures, infertility duration ≥ 3 years, chronic pelvic pain, and higher gravidity) were independently associated with concurrent EMS and CE. The overall EMS cohort showed lower clinical pregnancy rates and higher rates of persistent infertility and early pregnancy loss at 1 year than the control cohort; within the EMS cohort, concurrent CE was associated with poorer 1-year reproductive outcomes than EMS alone. However, this subgroup comparison was exploratory and unadjusted for potential confounders, and conclusions regarding the combined reproductive impact of the 2 conditions must await appropriately designed prospective studies.

Due to the retrospective design, causal inference or directional relationships cannot be established. Bidirectional inflammatory signaling, shared confounders, or coincidental coexistence may explain the observed association. The study sample is limited to surgically managed infertile EMS patients, and findings are not generalizable to asymptomatic populations. Future multicenter, large-scale prospective studies are needed to clarify temporal disease onset, shared molecular pathways, and standardized integrated diagnostic and therapeutic workflows for patients with concurrent EMS and CE.

## Author contributions

**Conceptualization:** HuiLiu Fan, Bei Liu, XiaoXia Wu, HongLan Wei.

**Data curation:** HuiLiu Fan, Bei Liu, XiaoXia Wu, HongLan Wei.

**Formal analysis:** HuiLiu Fan, Bei Liu, HongLan Wei.

**Funding acquisition:** HuiLiu Fan, Bei Liu, XiaoXia Wu, HongLan Wei.

**Investigation:** HuiLiu Fan, Bei Liu.

**Methodology:** HuiLiu Fan, Bei Liu, XiaoXia Wu.

**Project administration:** HuiLiu Fan.

**Resources:** HuiLiu Fan, HongLan Wei.

**Software:** HuiLiu Fan, XiaoXia Wu, HongLan Wei.

**Supervision:** HuiLiu Fan, XiaoXia Wu.

**Validation:** HuiLiu Fan, XiaoXia Wu, HongLan Wei.

**Visualization:** HuiLiu Fan, Bei Liu, XiaoXia Wu, HongLan Wei.

**Writing – original draft:** HuiLiu Fan, Bei Liu, HongLan Wei.

**Writing – review & editing:** HuiLiu Fan, Bei Liu, HongLan Wei.
